# Prevalence and antimicrobial susceptibility pattern of *Enterococcus* species isolated from different clinical samples at Black Lion Specialized Teaching Hospital, Addis Ababa, Ethiopia

**DOI:** 10.1186/s13104-018-3898-0

**Published:** 2018-11-06

**Authors:** Zelalem Tena Ferede, Kassu Desta Tullu, Solomon Gizaw Derese, Addisu Gize Yeshanew

**Affiliations:** 1 Department of Medical Laboratory Sciences, School of Allied Health Sciences, College of Health Sciences, Addis Ababa University, Addis Ababa, Ethiopia; 2 Department of Microbiology, St. Paul’s Hospital Millennium Medical College, P.O.Box 1271, Addis Ababa, Ethiopia

**Keywords:** *Enterococcus*, Nosocomial infection, Antibiotic susceptibility

## Abstract

**Objective:**

*Enterococci* which are parts of the normal intestinal flora are opportunistic human pathogens. Their increasing importance is largely due to their resistance to antimicrobials. So the aim this study was to determine the prevalence and antimicrobial pattern of *Enterococcus* spp.

**Result:**

From the total of 422 samples processed, 15 *Enterococcus* species were isolated. In this study, linezolid were the drug of choice for *Enterococcus* species, which showed 100% sensitive followed by vancomycin 93.3% sensitive. In contrast, highly resistance (80%) was observed for ampicillin followed by doxycycline (73.3%). All of isolated *Enterococci* were sensitive to linezolid, however, resistance was observed to common antibiotics. The presence of multidrug resistant *Enterococci* in our study should be considered as an alarm for *Enterococcal* infections.

## Introduction

*Enterococcus* species have become problem of the world as emerging nosocomial infection and multi drug resistance bacteria [1]. Among several species which belong to genus *Enterococcus*, *E. faecalis* is the most common isolate that has an association with 80–90% of human *Enterococcal* infection. *E. faecium* is also isolated from 10 to 15% of infections [2, 3]. Infections commonly caused by enterococci include urinary tract infections, bacteremia, endocarditis, catheter-related infections, wound and soft tissue infections, meningitis, respiratory infections, neonatal sepsis and intra-abdominal and pelvic infections [4, 5].

During the past decade, there has been a worldwide trend in increasing occurrence of *Enterococcus* species in the hospitals, a shift in the spectrum of *Enterococcal* infections and emergence of antimicrobial resistance among such isolates. This indicates that a need for continuous surveillance of the bacteria [6].

The second most frequent *Enterococcal* infections generally have been intra-abdominal and intra-pelvic abscesses or post-surgery wound infections [7]. In these settings, *Enterococcus* species are usually part of a mixed flora commonly found in the GIT so identifying the bacteria which cause the disease will be critical. Interactions among various bacteria have been demonstrated, and studies suggest that *Enterococcus* species can act synergistically with other bacteria to enhance infection. The third most frequent infection caused by these organisms is blood stream infections [1]. Other infections caused with lower frequency are central nervous system and neonatal infections. *Enterococcus* species rarely cause respiratory tract infections, osteomyelitis, or cellulitis [8].

*Enterococcus* species have become the second or third leading cause of nosocomial urinary tract infections (UTIs), wound infections (mostly surgical, decubitus ulcers, and burn wounds), and bacteremia in the United States [9, 10]. UTIs are the most common of the *Enterococcal* infections: *Enterococcus* species have been implicated in approximately 10% of all UTIs and in up to approximately 16% of nosocomial UTIs [11]. *Enterococcal* bacteremia is frequently associated with metastatic abscesses in multiple organs and high mortality rates. *Enterococcus* have also been considered an important cause of endocarditis; they are estimated to account for about 20% of the cases of native valve bacterial endocarditis and for about 6–7% of prosthetic valve endocarditis [12, 13]. Endocarditis remains among the most difficult to treat *Enterococcal* infections because of limitations on bactericidal antimicrobial therapy for *Enterococcal* infections, especially when caused by vancomycin-resistant *Enterococcus* (VRE). There is also a growing concern about the role of the *Enterococcus* species in endodontic and implant- and medical device-associated infections [8].

The emerging contest of *Enterococcus* nosocomial infection and resistance rates to the most common prescribed drugs vary considerably in different areas due to local antimicrobial prescribing practices, empirical treatment, and prevalence of circulating resistant *Enterococcus* species in a given area [14, 15]. The etiologies and their susceptibility to different antibiotics may have also changed over time. The irrational use of antibiotics in different set up has also immensely contributed to the antimicrobial resistance and emergence of multidrug resistant urinary isolates [16].

The emergence of nosocomial *Enterococcus* infection, multi drug resistance and VRE in different clinical sample were recorded in different country throughout the world. But, to our knowledge there was no previous data or study showing resistance pattern and prevalence of *Enterococcus* species in the study area. The aim of this study was to determine the prevalence of *Enterococcus* species and their antimicrobial susceptibility pattern isolated from different clinical samples in Black Lion Specialized Teaching Hospital, Addis Ababa, Ethiopia.

## Main text

### Methods

Cross-sectional was conducted at Black Lion Specialized Hospital from April to May 2016; this hospital is a one of the tertiary, teaching and national referral hospital in Ethiopia under the Federal Ministry of Health.

Patients who were seeking medical service at Black Lion Specialized Hospital during the study period considered as the source and all age groups who were suspected for UTI, septicemia, wound infection, endocarditic and meningitis, were the study populations.

Prevalence of *Enterococcus* species and their antimicrobial susceptibility pattern of *Enterococcus* species were dependent variables, whereas, socio-demographic variables, previous antibiotic usage, history of catheterization for urine sample, duration of admission and other associated risk factors, were independent variables.

#### Sample size and sampling technique

Since there is no previous study, prevalence of *Enterococcus* species in different clinical sample was calculated by using single population proportion formula taking, 50% prevalence. Therefore, a total of 422 study participants were included in this study. All consecutive *Enterococcal* infection suspected patients were included in the study.

#### Data collection and processing

Relevant information including; age, sex, source of infection, were gathered on a prepared questionnaires. Clinical samples (urine, blood, body fluid, CSF and Pus) were collected according to the microbiological sample collection techniques.

Prior to data collection, information about how to collect data was given to selected data collectors (two nurses and two laboratory technicians).

All clinical samples were collected by trained personnel with sterile container according to the standard operating procedures (SOP). All the samples were analyzed (examined) immediately arrived to the laboratory to ensure that the *Enterococcus* species present in the sample were isolated.

Blood for culture must be collected and dispensed with great care to avoid contamination of specimen and culture medium. After disinfecting the site of blood collection, 10–15 ml of blood for adult patient and 3–5 ml of blood for children were collected. Then all blood samples were cultured in blood culture bottles and incubated at 37 °C for 24 h to see hemolysis and turbidity.

Other specimens were cultured on blood and MacConkey agar media immediately and incubated at 37 °C for 24 h. Then, the suspected colony on the culture was identified primarily as *Enterococcus* spp. according to the colony morphology and gram staining.

Growth of enterococci on solid media were as follows: On blood agar plate, enterococci produced small, round, smooth, transparent colonies and some colonies showed hemolysis alpha(α) or beta(β) and some were non-hemolytic. On MacConkey’s medium, Enterococci produced tiny deep pink colonies.

#### Drug susceptibility patterns

The antimicrobial susceptibility testing was performed by using the Kirby Bauer disc diffusion method according to the National Committee for Clinical Laboratory Standards (NCCLS, 2014) guidelines.

The turbidity of suspension was determined in comparison with 0.5 MacFarland standards. A sterile swab was dipped in the broth suspension and excess suspension was removed by pressing the swab against the wall of the tube. The entire surface of MHA plate was uniformly flooded with suspensions and allowed to dry for about 15–30 min. The antimicrobial impregnated disks were placed by using sterile forceps at least 24 mm away from each other to avoid the overlapping zone of inhibition [17]. Grades of susceptibility pattern was recognized as sensitive, intermediate and resistant by comparison of zone of inhibition as indicated in the manufacturer’s guidance [17, 18]. The antibiotic susceptibility pattern was interpreted as per the manufacturer guide line [3].

#### Quality control

Standard operating procedures were strictly followed. Quality control was performed to check the quality of medium. Each new lot was quality controlled before use by testing the ATCC *E. faecalis* 29212, as per the Clinical and Laboratory Standards Institute guidelines standard strains [17]. All statistical analyses were performed with the SPSS statistical software package (version 20). Probability values (P) of < 0.05 were considered as statistically significant. Finally, the study findings were explained in words and tables.

#### Ethical considerations

Ethical clearance and permissions were obtained from the department of ethics committee of medical laboratory science in Addis Ababa University; and written informed consent was obtained from voluntary participants and parents or guardians for children under 18 years old during data collection. The findings of pathogenic bacteria were reported to the responsible body.

### Results

#### Socio-demographic characteristics of study participants

A total of 422 participants were involved in the study from those 41.71% were females and 58.29% were male participants. The mean ± SD age of participants was 23 ± 10 years (range 1–87) (Table 1).

**Table 1:** Socio-demographic characteristics of study participants (n = 422) among patients attending at Black Lion Specialized Hospital, Addis Ababa, Ethiopia, 2016

Characteristics	Frequency	Percent
Sex		
Male	246	58.29
Female	176	41.71
Residence		
Urban	245	58.0
Rural	177	42.0
Age group		
< 5	74	17.5
5–15	111	26.3
16–26	91	21.6
27–37	62	14.3
38–48	31	7.3
49–59	25	5.9
> 59	28	6.6
Educational status		
Illiterate	41	9.7
Under school age	107	25.4
Primary school	147	34.8
Secondary school	58	13.7
Diploma	35	8.3
Above diploma	34	8.1

#### *Enterococcus* isolates

Fifteen strains of *Enterococci* were isolated from a total of 422 various clinical specimens (Table 2).

**Table 2:** Prevalence of *Enterococcus* spp. in relation to different socio-demographic characters among patients attending Black Lion Specialized Hospital, Addis Ababa, Ethiopia, 2016

Characteristics	Frequency	Negative (%)	Positive (%)	X^2^	P value	OR
Sex						
Male	246	239 (97.6%)	7 (2.4%)	0.4	0.5	0.15
Female	176	168 (95.5%)	8 (4.5%)	0.4	0.5	1.6
Age group						
< 5	71	71 (1005)	0 (0%)	2.02	0.2	0.00
5–15	109	105 (96.3%)	4 (3.7%)	0.05	0.8	1.05
16–26	90	88 (97.8%)	2 (2.2%)	0.2	0.7	0.6
27–37	63	62 (98.41%)	1 (1.59%)	0.3	0.6	0.4
38–48	31	31 (100%)	0 (0%)	0.4	0.5	0.0
49–59	25	23 (92%)	2 (8%)	0.5	0.5	2.6
> 59	33	27 (81.8%)	6 (18.2%)	17.95	0.002	9.38
Specimen						
Urine	116	109 (93.9%)	7 (6.1%)	2.0	0.2	2.4
Blood	189	183 (96.8%)	6 (3.2%)			
Ascetic fluid	39	38 (97.4%)	1 (2.6%)			
Pus	47	46 (97.9%)	1 (2.1%)			
CSF	31	31 (100%)	0 (0%)			
Patient source						
Out patient	103	102 (99%)	1 (1%)		0.2	
Medical ward	164	154 (93.9%)	10 (6.1%)			
Pediatric ward	102	99 (97%)	3 (3%)			
Surgical ward	31	30 (96.8%)	1 (3.3%)			
Orthopedics	22	22 (100%)	0 (0%)			
Urine sample						
Catheterized	7	3 (42.9%)	4 (57.1%)	25.4	0.000002	47
Non catheterized	109	106 (97.2%)	3 (2.8%)			

#### Associated factors

Since *Enterococcus* species have now emerged as nosocomial pathogens, in this study from 15 of the *Enterococcus* spp. isolated 14 were form hospitalized patients and the higher prevalence, 13 of the 14 *Enterococcus* spp. were from patients which were stayed in the hospital from 3 to 6 days with statistical significance difference (P < 0.05).

#### Antibiotic susceptibility patterns

The most effective antibiotic for *Enterococcus* spp. was linezolid with 100% sensitivity (15/15), followed by vancomycin 13/15 (86.7%) (Table 3).

**Table 3:** Antibiotics resistance patterns of *Enterococcus* spp. at Black Lion Specialized Hospital, Addis Ababa, Ethiopia, 2016

Drugs	Resistance (%)	Sensitive (%)	Intermediate (%)
Ampicillin	12 (80%)	3 (20%)	0
Chloramphenicol	8 (53.3%)	7 (46.7%)	0
Gentamycin	9 (60%)	6 (40%)	0
Ciprofloxacin	8 (53.3%)	7 (46.7%)	0
Doxycycline	11 (73.3%)	4 (26.7%)	0
Linezolid	0	15 (100%)	0
Vancomycin	1 (6.7%)	14 (93.3%)	0

### Discussion

The epidemiology of *Enterococci* is not fully understood since there are striking differences among different species of resistant isolates obtained from various geographic locations [11]. Despite the fact that enterococci have been considered to be relatively low virulent in the past few years, they are among all nosocomial pathogens that have emerged as a significant concern [6, 19]. Intestinal colonization with resistant *Enterococcal* strains is more common than clinical infection. Colonized patients are a potential source for the spread of organisms to the health care workers, the environment and other patients [20].

In our study, the prevalence of *Enterococci* among different clinical samples was 3.5%. This prevalence rate was consistent with the findings of other authors who found the prevalence rate in Egypt (3.3%), in Bangladesh (3.2%), in India (2.3%) and in Asian pacific (3.6%) [21–23]. On the other hand, the prevalence in the present study was higher than the report from Kenya (0.22%) [24], and different hospitals of Ethiopia including Jimma, Felege Hiwot and University of Gondar Teaching Hospital that accounted 0.59%, 0.64% and 2.13% respectively [10, 25, 26]. However, it was lower than the prevalence study done in USA and Canada, 18.0% and 21.2%, respectively [21].

The variation in results might be explained due to the different characteristics in the study participants and in the use of conventional methods for identification of *Enterococci*. As the opinion explained earlier by other study [13, 27], it was mentioned that the conventional methods for the identification of microorganisms are based on phenotypic and culture characteristics and may not be able to identify the causative organism correctly when strains with unusual phenotypes. Moreover, the study subjects included in most of the previous studies were hospitalized patients as their aim was to show hospital acquired infections. The gradual increase in the prevalence of enterococci infections might have also contributed to the increased prevalence as evidenced by other studies.

All *Enterococcus* spp. isolates, in the present study, were sensitive to linezolid. This result was in agreement with studies in India, Bangladesh and India which reported that none of the *Enterococci* isolates were resistant to linezolid [28, 29].

In general, only one *Enterococcus* species were sensitive to all drugs used in our study but the other isolates were resistant to at least one drug. 66.7% of the isolated *Enterococci* were multi drug resistances which were resistant to three and above drugs. This result was comparable with the study reports in Egypt and Nigeria which accounts [30, 31].

### Conclusion

The study showed that the rate of isolated *Enterococci* had variable degrees of resistance to the antibiotics, but all were sensitive to linezolid by disc diffusion methods. The presence of high percentage multidrug resistant *Enterococci* in our study should be considered as alarm and further study in large scale is needed.

## Limitation of the study

This study has limitation to identify the isolated *Enterococci* up to species level.

## Abbreviations

BSI: blood stream infection
CDC: Center for Disease Control program
CLSI: Clinical Laboratory Standard Institute
CNS: central nervous system
GIT: gastro intestinal tract
HIV: human immuno deficiency virus
HLAR: high level aminoglycoside resistance
HLGR: high level gentamicin resistance
LAP: lucien amino peptides
MIC: minimum inhibitory concentration
MSU: mid-stream urine
QC: quality control
RNA: ribo nucleic acid
SOP: standard operating procedure
SPP: species
SPSS: statistical package for social science
UTI: urinary tract infection
VRE: vancomycin resistance Enterococcus
WHO: World Health Organization
